# Heparanase and the hallmarks of cancer

**DOI:** 10.1186/s12967-020-02624-1

**Published:** 2020-11-30

**Authors:** Krishnath M. Jayatilleke, Mark D. Hulett

**Affiliations:** Department of Biochemistry and Genetics, La Trobe Institute for Molecular Science, La Trobe University, Plenty Road & Kingsbury Drive, Melbourne, VIC 3086 Australia

**Keywords:** Heparanase, Cancer, Hallmarks of cancer, Extracellular matrix, Tumour microenvironment

## Abstract

Heparanase is the only mammalian enzyme that cleaves heparan sulphate, an important component of the extracellular matrix. This leads to the remodelling of the extracellular matrix, whilst liberating growth factors and cytokines bound to heparan sulphate. This in turn promotes both physiological and pathological processes such as angiogenesis, immune cell migration, inflammation, wound healing and metastasis. Furthermore, heparanase exhibits non-enzymatic actions in cell signalling and in regulating gene expression. Cancer is underpinned by key characteristic features that promote malignant growth and disease progression, collectively termed the ‘hallmarks of cancer’. Essentially, all cancers examined to date have been reported to overexpress heparanase, leading to enhanced tumour growth and metastasis with concomitant poor patient survival. With its multiple roles within the tumour microenvironment, heparanase has been demonstrated to regulate each of these hallmark features, in turn highlighting the need for heparanase-targeted therapies. However, recent discoveries which demonstrated that heparanase can also regulate vital anti-tumour mechanisms have cast doubt on this approach. This review will explore the myriad ways by which heparanase functions as a key regulator of the hallmarks of cancer and will highlight its role as a major component within the tumour microenvironment. The dual role of heparanase within the tumour microenvironment, however, emphasises the need for further investigation into defining its precise mechanism of action in different cancer settings.

## Background

The common defining feature of all cancers is the loss of cellular regulation mechanisms through genetic changes leading to uncontrolled cell division, resulting in either benign or malignant neoplasms. A number of common characteristic features termed the ‘hallmarks of cancer’ were first described by Hanahan and Weinberg [1]. Six hallmarks were initially proposed as sustaining proliferative signalling, evading growth suppressors, resisting cell death, enabling replicative immortality, inducing angiogenesis and activating invasion and metastasis. These are now accompanied by four additional ‘enabling characteristics’ and ‘emerging hallmarks’, namely genome instability and mutation, tumour-promoting inflammation, reprogramming energy metabolism and avoiding immune destruction [2].

The extracellular matrix (ECM) is essential for tissue integrity and homeostasis. Heparan sulphate (HS) is an important component of the ECM by contributing to maintenance of its structural integrity and regulatory functions in the form of heparan sulphate proteoglycans (HSPGs). HSPGs exist in a variety of forms in the ECM and basement membrane (BM; perlecan, agrin and collagen XVIII) as well as on cell surfaces (syndecans and glypicans) and intracellularly (serglycin) [3]. Additionally, HS sequesters a number of growth-promoting and signalling molecules, collectively termed HS-binding proteins (HSBPs), thus regulating their bioavailability and functions [3–6].

The physiological expression of heparanase (HPSE) is limited to a few cell and tissue types such as platelets, immune cells, and the placenta [7–11]. The enzymatic activity of HPSE leads to ECM remodelling and the increased bioavailability of HSBPs sequestered on HS chains [4]. Under physiological conditions, the expression of HPSE is strictly regulated to prevent non-specific tissue damage [12–18]. Dysregulated gene expression, a key hallmark of cancer, drives the overexpression of HPSE in the tumour microenvironment (TME), leading to pathological ECM remodelling and the liberation of cancer-promoting HSBPs [2, 19]. HPSE also exhibits a variety of non-enzymatic functions such as regulating gene expression, promoting cell adhesion and tumour-promoting pro-coagulant activity [20, 21]. The overexpression of HPSE in cancer thus enhances tumour growth and metastasis, resulting in a poor clinical prognosis [21, 22]. The subsequent sections will discuss the mechanisms by which HPSE regulates each of the hallmarks of cancer, which define it as a key component within the TME. Additionally, this review will also explore the complexities associated with utilising HPSE as an anti-cancer therapeutic target, considering its role in both physiological and pathological settings.

### The role of heparanase in the hallmarks of cancer

**Fig. 1:**
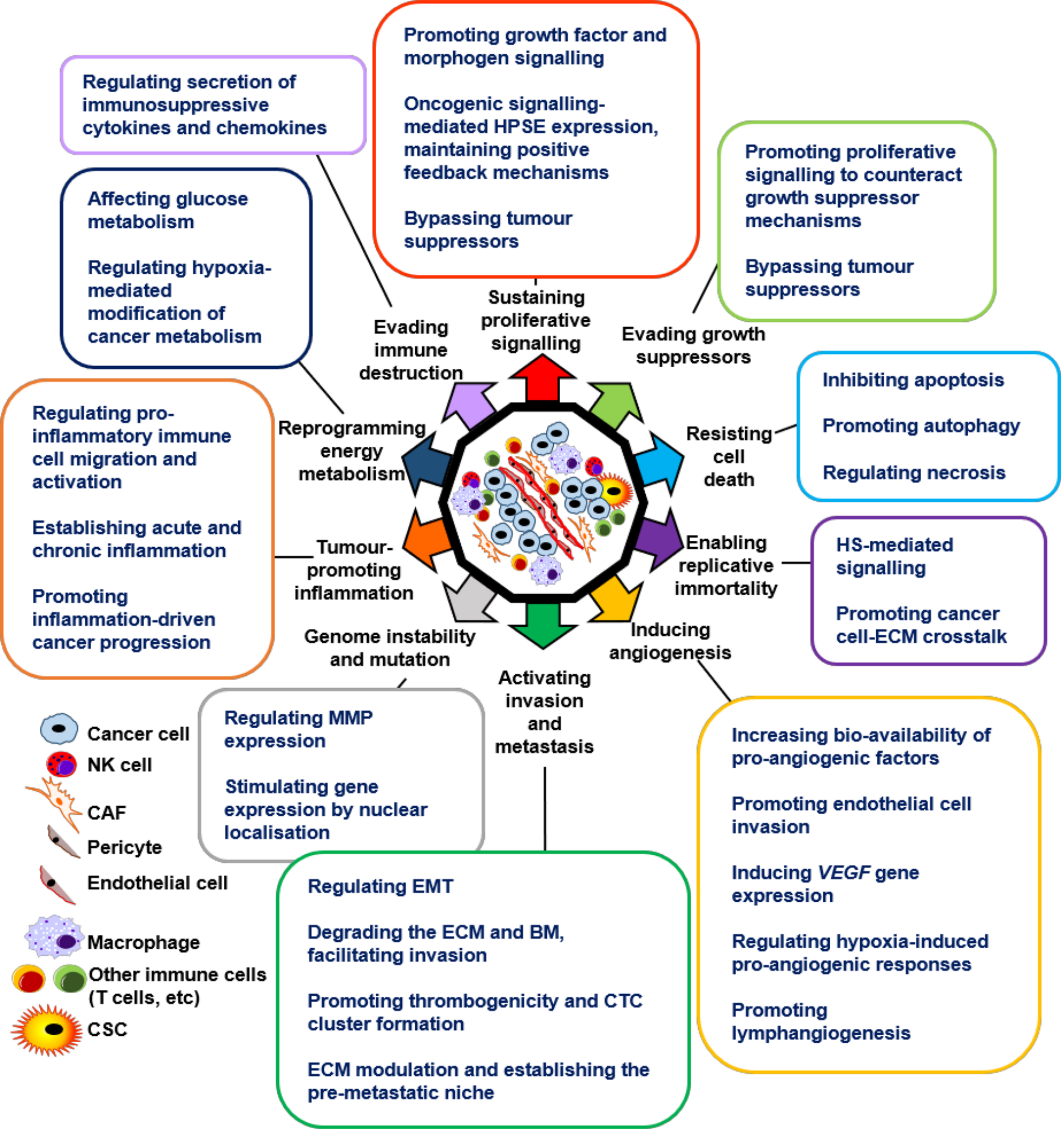
HPSE regulates all hallmarks and enabling characteristics of cancer. The enzymatic and non-enzymatic activity of HPSE regulates the classic and emerging hallmarks of cancer as well as all enabling characteristics. The key mechanisms of action undertaken by HPSE in facilitating each of the hallmarks and characteristic features are listed. By virtue of its multi-faceted nature, HPSE has emerged as a key regulatory component within the TME

HPSE regulates the classic and emerging hallmarks of cancer as well as all enabling characteristics (Fig. 1), as discussed in the following sections.

#### 1. Sustaining proliferative signalling

Cellular proliferation is a meticulously choreographed process which is dysregulated in cancers [23]. HS binds to and sequesters a variety of HSBPs which regulate cellular proliferation, in turn restricting their bioavailability and governing downstream signal transduction. Remodelling of the ECM through HPSE-mediated HS cleavage liberates these HSBPs, thus upregulating cellular proliferation [4, 24]. Several key HSBPs and their relationships with HPSE are highlighted below.

Fibroblast growth factor (FGF): The binding of FGF to HS is vital for dimerization and signalling through the FGF-receptor (FGFR) [25, 26]. The overexpression of HPSE in mouse organs and human tumours has been shown to correlate with enhanced 6-O-sulphation of HS, which promoted the formation of ternary complexes with FGF-1 or -2 and FGFR [27]. Hepatocyte growth factor (HGF): HGF-mediated c-MET signalling is observed in cancer with its expression correlating with that of HS [28–30]. HPSE activity enhances HGF expression and signalling through syndecan shedding. It has also been shown that HGF activates the phosphatidylinositol-3-kinase/protein kinase-B (PI3K/Akt) and nuclear factor kappa-light-chain-enhancer of activated B cells (NF-κB) signalling to promote HPSE in cancer cells, resulting in a poor clinical prognosis in gastric tumours [31]. Vascular endothelial growth factor (VEGF): HPSE expression in the TME is directly related to the release of HS-bound VEGF [32]. The VEGF family members are well known for regulating angiogenesis, vascular permeability and lymphangiogenesis. The expression of HPSE has also been shown to promote the expression of VEGF in a Src-dependent manner [33]. Furthermore, VEGF can influence the expression levels of HPSE, demonstrating a synergy between HPSE and VEGF in cancer [34]. Epidermal growth factor (EGF): EGF-receptor (EGFR)-mediated signalling is a potent driver of the cell cycle, enhancing proliferation and is implicated in numerous cancer settings [35–37]. HPSE activates EGFR signalling through HS (specifically, syndecan) cleavage and promotes chemotherapy resistance in colorectal cancer [38]. The expression of heparin-binding EGF-like growth factor with a high affinity to HS correlates with HPSE expression, suggesting a HPSE-driven regulation of EGF expression [39]. In brain-metastatic breast cancer, EGF induces the nucleolar localisation of HPSE, resulting in deoxyribonucleic acid (DNA) topoisomerase-I modulation and enhanced proliferation [40]. Furthermore, both enzymatically active and inactive HPSE trigger pathways that lead to EGFR phosphorylation, which correlated with head and neck cancer progression [41]. Transforming growth factor (TGF)-β: TGF-β plays a complicated role in cancer cell proliferation, initially as a tumour suppressor in early tumorigenesis, before transitioning to a tumour promoter in later stages [42, 43]. TGF-β has been shown to interact with HS, which regulates its bioavailability and signalling capacity [44, 45]. Although Batool et al. showed that overexpressing HPSE attenuated TGF-β signalling, others have demonstrated a positive correlation, suggesting the upregulated HPSE expression and invasive potential upon TGF-β treatment [46–48]. Hedgehog (Hh): Hh-mediated signalling has been shown to correlate directly with cell cycle regulation [49]. The Hh pathway can be modulated in some settings by HS where the binding of Hh to HS followed by its release upon HPSE activity can lead to increased Hh signalling and an aggressive cancer phenotype [50–54]. Wnt: HPSE has been shown to mediate Wnt signalling in cancer settings via studies on medulloblastoma and pancreatic cancer [55, 56].

#### Oncogenic signalling incorporating HPSE and positive feedback mechanisms

HPSE acts in concert with a number of oncogenes such as *Ras*, *Myc* and *BRAF*, which promotes tumour growth. A correlation between HPSE and Ras expression was demonstrated in driving tumorigenesis in murine models of breast and skin cancer [57]. Although a relationship similar to that between Ras and HPSE has not been reported for Myc, human telomerase reverse transcriptase (hTERT), which plays a pivotal role in maintaining telomere length in many cancers, was shown to correlate with Myc and HPSE expression in gastric cancer [58]. Expression of Myc driven by hTERT in turn activates further hTERT transcription and HPSE expression, leading to downstream tumour-promoting enzymatic activity [58]. B-Raf kinase, the product of the mutant *BRAF* oncogene upregulates HPSE expression through *HPSE* promoter activation [18].

Disrupting negative-feedback mechanisms that attenuate proliferative signalling enables cancer progression [2]. HPSE-regulated growth factors such as HGF, VEGF and TGF-β not only promote tumour growth, but can also upregulate HPSE expression [31, 34, 46]. This maintains a constant positive feedback loop, driving both HPSE expression and its resultant downstream effects. The phosphatase and tensin homolog (PTEN) is a potent tumour suppressor, de-phosphorylating phosphatidylinositol-(3,4,5)-trisphosphate and counteracting PI3K/Akt activity [59]. Partial or complete PTEN inactivation is associated with a large proportion of cancers [60]. The non-enzymatic activity of HPSE in stimulating the PI3K/Akt pathway was demonstrated in endothelial cells [61]. A later observation of integrin-dependent PI3K/Akt activation following the binding of HPSE to a cell surface receptor further highlighted the non-enzymatic activity of HPSE in promoting tumour signalling [62]. Additionally, the activation of the PI3K/Akt pathway by HGF signalling was shown to stimulate the downstream expression of HPSE, promoting gastric cancer metastasis [31]. These data suggest that HPSE may be able to bypass PTEN-mediated tumour suppression, by directly influencing the PI3K/Akt pathway which in turn may upregulate HPSE expression.

#### 2. Evading growth suppressors

HPSE-driven mechanisms overlap in their promotion of proliferative signalling as well as evading growth suppressors. A key regulator of cancerous cell growth is the *TP53-*encoded p53 tumour suppressor [63, 64]. Although HPSE plays no role in causing *TP53* gene mutations, HPSE expression is regulated by wild-type p53 binding to the *HPSE* promoter [14]. *TP53* gene mutations lead to upregulated HPSE expression, which promotes a number of HPSE-mediated growth suppressor-evasion mechanisms.

The ability of HPSE to activate PI3K/Akt in a non-enzymatic manner, essentially bypassing PTEN signalling as previously discussed, is evidence of its ability to counter tumour-suppressive mechanisms [62]. Another, although controversial tumour suppressor is the signal transducer and activator of transcription (STAT) family protein member STAT3 [65]. In a study of head and neck cancer, HPSE was shown to induce the phosphorylation of STAT3 through Src and EGFR phosphorylation, leading to a poor clinical outcome [66].

In support of its tumour suppressive role, a number of studies have demonstrated that the lack of TGF-β signalling promoted tumour growth [67–70]. SMAD-family-member-4, a component of the TGF-β signalling pathway was shown to inhibit HPSE activity, suggesting the tumour-suppressive role of TGF-β [71]. It can therefore be argued that by regulating other signalling pathways that do promote tumour growth, HPSE may effectively be bypassing the tumour-suppressive role of TGF-β.

#### 3. Resisting cell death

##### HPSE inhibits apoptosis

Apoptosis, or programmed cell death was discovered as a fundamental biological process in maintaining tissue homeostasis and occurs in response to a number of stimuli [72, 73]. Unlike healthy cells, cancer cells are under constant stress brought about by processes such as genomic instability and hypoxia but have evolved means to inactivate apoptosis that is normally triggered under such conditions.

The anti-apoptotic role of HPSE can be attributed largely to its ability to promote and sustain tumour growth via HS-mediated signalling [4]. HPSE-promoted release of FGF has been shown to inhibit apoptosis in breast cancer cells and prolong tumour survival [74]. Basic FGF is known to inhibit caspase-3 and in turn, downregulate apoptosis [75]. Additionally, the non-enzymatic activity of HPSE in activating Akt was shown to inhibit oxidative-stress and growth factor starvation-induced apoptosis [62]. HPSE further facilitates the activation of Src [33]. Activated Src has been shown to suppress apoptosis by mechanisms such as the degradation of Bik, a BH3-only protein and through the phosphorylation of the apoptosis suppressor Ku70 [76, 77].

*HPSE* gene silencing showed that its inactivation induces apoptosis in pituitary tumour cells with an observed increase in sub-G1 events and poly adenosine diphosphate ribose polymerase cleavage [78]. The drug-mediated inhibition of HPSE has also been demonstrated to promote apoptosis in cancer cells, further validating its anti-apoptotic role. Inhibition of HPSE with PG545, a HS-mimetic, promoted apoptosis in pancreatic cancer cells [32]. Treatment with yet another HS-mimetic PI-88, promoted tumour apoptosis in RIP1/Tag2 transgenic mice, which present a multi-step process of islet cell carcinoma [79, 80].

##### HPSE-mediated autophagy

Mammalian autophagy is a well-characterised process with both physiological and pathological functions [81, 82]. The induction of autophagy was initially thought to inhibit tumorigenesis, suggesting a cytoprotective role [83]. However, autophagy has been shown to enable cancer cell survival and lead to chemoresistance [84–86]. HPSE has been shown to reside within lysosomes, suggesting a possible involvement in autophagy [87]. A key regulator of autophagy is the mammalian target of rapamycin-1 (mTOR1) [88]. HPSE expression was shown to reduce mTOR1 activity, which promoted autophagy, thus enhancing tumour growth and chemoresistance [89]. Shteingauz et al*.* further demonstrated that the inhibition of autophagy and HPSE resulted in reduced tumour growth, suggesting a potential therapeutic strategy. Therefore, in a rather interesting twist, the intracellular activity of HPSE is suggested to mediate tumour cell survival through promoting autophagy, a mechanism designed to maintain cellular homeostasis.

##### HPSE and necrosis

There is no clear evidence for the direct involvement of HPSE in tumour necrosis. However, HPSE has been shown to regulate tumour necrosis factor (TNF) expression, a key enabler of necrosis, whose superfamily members possess pro-tumorigenic and pro-tumour inflammatory activity [90–94]. Tumour-associated macrophages (TAMs) produce TNF-α in a HPSE-dependent manner, driving inflammation and tumour growth [10]. HPSE has been shown to regulate necrosis in several other disease settings. In a study of patients with diabetic foot necrosis, HPSE-driven post-surgical pro-coagulant activity predicted a successful clinical outcome, whereas a reduction of such predicted necrosis [95]. In a study of osteonecrosis, an increased level of HPSE was shown to promote the destruction of the femur head [96].

#### 4. Enabling replicative immortality

Cancer cells by definition, are immortal. Telomeres at the ends of chromosomes are key in regulating cellular replication, and the expression of telomerase by cancers enables replicative immortality [97–100]. As previously described, a synergistic relationship between telomerase and HPSE may exist [58].

FGF is a key growth factor in the inhibition of cellular senescence and the promotion of cancer [25, 101, 102]. Tumour-induced HPSE expression was shown to regulate HS biosynthesis and promote FGF activity, leading to enhanced tumour growth [27]. HS has also been shown to play a key role in FGF signalling by increasing its radius of diffusion [103]. Furthermore, HS fine-tunes the FGFR signalling pathway through variable sulfation, thereby overcoming cellular senescence [104].

The interaction between cancer cells and the ECM is also key to maintaining immortality and overcoming growth-inhibitory signals. Integrins are a major cell-ECM adhesive molecule expressed by both healthy and cancerous cells, thus enabling cell-ECM communication [105]. Cancer stem cells (CSCs), first described in acute myeloid leukaemia, are known to play a critical role in the initiation and maintenance of tumours [106, 107]. Studies have shown that integrins play a key role in the maintenance of CSCs, with HS suggested to promote cell-ECM adhesion by interacting with integrins [105, 108]. Integrin-mediated cellular adhesion via αVβ3 and α5β1 was shown to promote HPSE-induced Akt phosphorylation and the induction of the pro-survival PI3K/Akt pathway [62]. Interestingly, integrin α5β1 has been demonstrated to be a facultative proteoglycan [109]. This multi-faceted relationship between HPSE, HS and integrins could enable tumour growth, with HPSE playing a limited but important role in enabling replicative immortality.

#### 5. Inducing angiogenesis

Tumour-associated neovasculature is the result of engaging an ‘angiogenic switch’, causing quiescent vasculature to sprout new vessels continuously [110]. In addition to sustaining growth of the primary tumour, angiogenesis promotes metastasis by providing a means of escape for cancer cells [111]. VEGF is a prominent HSBP, with VEGF-A as the major pro-angiogenic VEGF family member, constituting the prime focus of this hallmark [112–114]. FGF has also been demonstrated as a potent regulator of angiogenesis, with numerous studies demonstrating that FGF is key in tumours developing resistance to VEGF inhibition [25, 115, 116]. The enzymatic activity of HPSE promotes tumour angiogenesis via the activation of the VEGF and FGF signalling pathways through HS cleavage [4]. Numerous studies using pre-clinical disease models and patient tumour samples have demonstrated the key role of HPSE in activating the angiogenic switch and promoting this hallmark, as highlighted below.

A strong correlation between the expression of HPSE and microvessel density was observed in tumour samples of endometrial cancer patients, which correlated with highly aggressive tumours [117]. HPSE-overexpressing MCF-7 human breast cancer cells showed increased angiogenesis in vivo and correlated with large tumour size [74]. Histological analysis of human colorectal cancers showed a positive correlation between HPSE expression and tumour angiogenesis [118]. Endothelial cells exhibit an invasive phenotype at the onset of angiogenesis, as well as atherosclerosis and wound healing, which was shown to be mediated by HPSE [119]. HPSE expression in myeloma cells enhances syndecan-1 shedding through activation of MMP-9 [120]. Interestingly, Akt phosphorylation in endothelial cells was mediated by HPSE in a non-enzymatic manner which resulted in endothelial cell migration and invasion [61]. The silencing of HPSE expression resulted in a reduction of angiogenesis in an in vivo model of lymphoma, which prolonged survival [121]. A second study silencing HPSE expression in the MDA-MB-435 human breast cancer cell line demonstrated a similar effect on angiogenesis [122]. The combined effects of HPSE and cyclooxygenase-2 (COX-2) in promoting tumour angiogenesis was demonstrated in human oesophageal cancer patients, with an increased HPSE expression leading to poor survival [123]. In addition to liberating HS-bound VEGF, HPSE was shown to induce the expression of VEGF in correlation with p38 phosphorylation and Src activation, which promoted angiogenesis in vivo in an MDA-MB-435 xenograft model [33]. This suggests that the expression of HPSE may correlate with *VEGF* gene regulation.

Several other studies have demonstrated that the inhibition of HPSE leads to the inhibition of angiogenesis, enhancing survival. Treatment with PI88, a potent small molecule inhibitor of HPSE, inhibited angiogenesis in vitro and in vivo in a model of rat adenocarcinoma, resulting in impaired tumour growth [124]. The PG500 series of HS mimetics were developed as potential HPSE inhibitors for clinical use [125]. The lead drug candidate, PG545, was shown to bind VEGF and FGF and effectively reduce angiogenesis in vitro and affect in vivo tumour development. Further pre-clinical studies with PG545 demonstrated its anti-angiogenic effects in vivo, resulting in increased survival [126, 127]. Additionally, a low molecular weight heparin derivative was also shown to inhibit tumour angiogenesis in vivo, as well as λ-carrageenan, a HS-mimetic [128, 129].

##### HPSE and immune cell-driven angiogenesis

The infiltration of solid tumours by immune cells is well-characterised [130]. Infiltrating immune cells could at times be detrimental to the tumour, but in many cases can sustain its development. The pro-angiogenic effects of tumour-associated immune cells such as macrophages, neutrophils, myeloid-derived suppressor cells and mast cells have been reported in a number of studies [131–134]. HPSE is produced by a variety of immune cells and has been demonstrated in its capacity to activate and regulate the function and migration of a number of immune cell populations [7, 10, 11, 135–138]. This raises the possibility that tumour-associated immune cells may enhance angiogenesis by virtue of their HPSE- expression capacity and HPSE-mediated activation.

##### HPSE and hypoxia

The phenomenon of tumour hypoxia, the various adaptations by solid tumours to overcome oxygen starvation and the implications of hypoxia to patient survival are well characterised [139, 140]. Cells respond to hypoxia by expressing hypoxia-inducible factors (HIFs), which promote survival [141, 142]. On this account, HIFs have generated much interest as cancer therapeutic targets [141, 143–145].

Cancer cells exposed to hypoxic conditions were shown to upregulate HPSE expression in an NFκB-dependent manner [146]. COX-2 was shown to be a key component in HPSE-mediated HIF-1α expression, leading to increased tumour angiogenesis [147]. Hypoxia was further shown to not only promote angiogenesis, but also to promote invasion in a HPSE-dependent manner [148]. HPSE was also shown to play a role in radiation resistance by upregulating the HIF-1 pathway with correlated upregulation of both VEGF and FGF [149].

##### HPSE and lymphangiogenesis

Lymphangiogenesis and the dynamic role of tumour-associated lymphatic vessels in the TME and in the metastatic cascade are well understood [150]. FGF-2, VEGF-C and VEGF-D are prominent regulators of lymphangiogenesis and enhance the metastatic spread of tumours, generating clinical interest [151–153]. FGF and VEGF family members are sequestered by HS within the TME, with HPSE facilitating their release and activity [154, 155].

The relationship between HPSE expression, lymphangiogenesis and overall tumour grade has been demonstrated in a number of studies. In a pre-clinical model of inflammation in rats, HPSE expression by neutrophils was shown to regulate lymphangiogenesis via the enhanced bioavailability of VEGF-A [156]. Furthermore, in clinical studies of lung, pancreatic and head and neck cancer patients, HPSE expression upregulated VEGF-C signalling and was shown to promote invasion [157–159]. A relationship between COX-2 and lymphangiogenesis has also been demonstrated in a study of breast cancer patients, whereby COX-2 expression correlated with that of VEGF-C, promoting lymph node metastasis [160]. In a later study of cervical cancer patients, this relationship was more closely examined and it was demonstrated that HPSE promoted the expression of COX-2, leading to VEGF-C signalling [161].

#### 6. Activating invasion and metastasis

The most formidable hallmark of a cancer is its ability to activate invasion and metastasis, which is responsible for the majority of cancer deaths [162]. Metastasis is a complex, multi-step, non-random process resulting in the dissemination of malignant cells from its origin to distant sites [163]. Initially considered a late event in tumour progression, it is now evident that invasion and metastasis can occur relatively early [164]. The ‘seed and soil hypothesis’ proposed by Stephen Paget provided an early insight into metastasis [165]. This revealed a distinct relationship between metastatic tumour cells (seeds) and the metastatic microenvironment (soil) and described metastasis as a targeted process. Current treatment options face numerous challenges when targeting metastatic disease, which poses a major clinical challenge [166].

For the purpose of this review, the metastasis of epithelial carcinomas will be considered. Cancer cells disseminate from the primary tumour by gaining invasive capabilities. This is enabled by the adoption of mesenchymal features, in a process known as ‘epithelial-mesenchymal transition (EMT)’ [167]. This is driven by transcription factors such as Snail, Slug, Zeb1 and Twist, leading to cytoskeletal reorganisation, loss of cell–cell junctions, loss of apical-basal polarity with the gain of a front-rear polarity, changes in cell shape and gene expression and acquiring the ability to degrade ECM components [168]. Recent data suggest a ‘partial-EMT’ phenotype in metastatic cells, rather than a fully mesenchymal state which may enhance metastatic colonisation [169]. Several studies have shown that HPSE is able to induce EMT in disease settings such as myeloma and renal injury [170, 171]. Additionally, the inhibition of HPSE has been shown to block mesenchymal features both in vitro and in vivo [170]. Furthermore, the sulodexide-mediated inhibition of HPSE controls EMT-driven tubular fibrosis in a diabetic nephropathy setting [172]. A key regulator of EMT is FGF, whose signalling pathway is activated by HPSE, leading to the promotion of EMT [27, 173]. TGF-β is also a potent regulator of EMT, shown to promote renal fibrosis and cancer [174, 175]. HPSE is a key player in TGF-β-mediated EMT, further solidifying its role in promoting this vital pro-metastatic phenotype [176].

A significant rate-limiting step in the multi-step metastatic cascade is the migration of tumour cells through the ECM, which acts as a physical barrier. Indeed, the degradation of HS has been shown to be a key component in tumour cell invasion [7]. The members of the matrix metalloproteinase (MMP) along with the serine, aspartic and cysteine protease families are vital in invasion-promoting ECM disassembly [177–179]. The collective expression of ECM-degrading enzymes and HPSE at the invasive tumour front enables invading cells to effectively navigate through the ECM [180, 181]. Furthermore, the ability of HPSE to stimulate the expression of MMP-9 through extracellular signal-regulated kinase phosphorylation in a myeloma setting demonstrated its regulatory role in promoting invasion [182]. A number of clinical studies have demonstrated that the expression of HPSE at the tumour invasion front leads to a poor patient prognosis [183–186]. Recent studies have demonstrated that tumour hypoxia promotes the invasion of tumour cells via a number of mechanisms such as macrophage-driven signalling, acquisition of EMT features, increasing lysyl oxidase expression, enhanced Notch and mitogen-activated protein kinase activity and the expression of the *met* proto-oncogene [187–192]. As a regulator of hypoxia, HPSE can be suggested to promote hypoxia-driven metastasis in certain cancer settings.

The expression of HPSE is not only confined to tumour cells, but to other cell types in the TME as well. In an in vivo model of lymphoma, the TME was shown to contribute to HPSE activity of tumour xenografts, suggesting that host cells in the TME played an active role in HPSE expression of the primary tumour [193]. Neutralisation of HPSE activity within the TME affected primary tumour growth, indicating a bidirectional relationship between the tumour and its immediate environment with regards to HPSE expression. Furthermore, tumour-associated immune cells express HPSE [7, 10, 11, 135–137]. These observations collectively suggest that HPSE contributed by non-tumour components of the TME may also play a crucial role in the initial invasive stage of metastasis.

Invading tumour cells intravasate into the circulatory system either directly or via the lymphatic network, becoming circulating tumour cells (CTCs) [194]. Intravasation is a significant rate-limiting step of the metastatic cascade. Invasion through the ECM and in particular, the BM, a highly complex form of the ECM, are critical in intravasation with tumour cells employing various strategies to overcome these physical barriers [195, 196]. The role of proteases, in particular MMPs, in tumour invasion and intravasation are paramount [197, 198]. HPSE, with its aforementioned roles in stimulating angiogenesis and lymphangiogenesis, thereby actively participating in creating a vessel network for metastatic tumour cells and in degrading the ECM and BM, facilitating invasion followed by intravasation, is a major regulator of this crucial step of the metastatic cascade.

Once in circulation, CTCs face challenges of oxidative stress, shear force and immune destruction, resulting in approximately 0.01% of CTCs capable of forming metastases [199]. To overcome some of these challenges, CTCs are coated with platelets, mediated by tissue factor (TF) expressed on the CTC surface [200]. Platelets ‘cloak’ CTCs and form a physical barrier, which protects against shear force and masks CTCs against immune detection. The secretion of PDGF and TGF-β by platelets inhibit natural killer (NK) cell activity and sustain EMT pathways in CTCs [201–203]. CTCs can also interact with neutrophils which promote tumour cell survival and extravasation [204]. Neutrophils impart immunosuppressive functions by suppressing NK cell activity, as well as secrete MMPs, that enhance extravasation. The formation of neutrophil extracellular DNA traps designed to immobilise pathogens, trap and collect CTCs, promoting intraluminal survival [205]. The therapeutic potential of targeting of adhesion molecules that maintain CTC clusters has therefore been addressed to prevent metastatic colonisation [206]. In addition to its enzymatic means of promoting aspects of the metastatic cascade, HPSE has been shown to promote cellular adhesion by non-enzymatic means, with significant implications in CTC cluster formation [207]. HPSE in platelets has been shown to enhance their adhesive capacity, promoting thrombogenicity, which in turn supports CTC clusters [208]. The expression of HPSE in CTCs induces focal adhesion kinase and intercellular adhesion-molecule-1-mediated adhesion, enhancing metastasis in human breast cancer cells and was also shown to affect the adhesive properties of human glioma cells [209, 210]. The brain-metastatic potential of breast cancer CTCs isolated from patients was shown to be related to HPSE expression, a key component of the ‘metastatic signature’ of these cells [211]. This HPSE-mediated adhesiveness not only promotes CTC survival en route to distant sites, but also promotes extravasation and the eventual formation of the pre-metastatic niche.

Extravasation occurs with CTCs breaching the capillary wall at a distant site to form metastatic colonies, which concludes the ‘metastatic cascade’ [212]. Metastatic cells undergo trans-endothelial migration (TEM) at the extravasation site by the secretion of proteins that aid in disrupting vascular integrity, such as angiopoietin-like-4, VEGF and MMPs [213]. HPSE too, plays a key role in this process. As previously mentioned, the ability of HPSE to mediate cellular adhesion would aid in the attachment of CTCs to endothelial cells at the sites of extravasation [207, 214]. The sub-endothelial ECM degradation by HPSE has been shown to promote extravasation of immune cells such as mast cells, macrophages, neutrophils, therapeutic chimeric antigen receptor T (CAR-T) cells as well as tumour cells [7, 215–218].

CTC-associated platelets secrete nucleotides, which together with tumour cell-secreted chemokine (C–C motif) ligand-2 (CCL2) activate endothelial cells, rendering capillary walls permeable, promoting TEM [219, 220]. CCL2 recruits inflammatory monocytes which may differentiate into metastasis-associated macrophages and promote metastatic seeding [221]. HPSE has been shown to promote the activity of TAMs, which could suggest a role in aiding metastatic seeding [10].

The metastatic cascade concludes with colonisation. This depends on the receptive tissue microenvironment which can be prepared by the primary tumour, forming the ‘pre-metastatic niche’ [222]. Tumour-derived exosomes are implicated in the intracellular communication within the TME as well as the pre-metastatic niche formation in a number of cancer settings [223–225]. Studies have shown that HPSE activates the syndecan-syntenin-ALIX exosome pathway and that it is a key regulator of tumour-derived exosomes [226–228]. Interestingly, in a study of myeloma, it was demonstrated that chemotherapy stimulated the release of exosomes containing high HPSE levels that promoted cancer progression, indicating a role of HPSE in mediating resistance to cancer therapy [229]. The formation of the pre-metastatic niche involves significant remodelling of the existing ECM, which may be aided by HPSE contained within tumour-derived exosomes as well as HPSE produced by newly-arrived metastatic cells [230].

Metastatic outgrowths are highly reliant on the stromal microenvironment, similar to primary tumours [231]. Recently-arrived tumour cells may undergo dormancy, either failing to encounter a supportive stroma or experiencing suppressive cues [232, 233]. Dormant tumour cells reside in specialised niches and may acquire stem cell traits, which are a prerequisite for eventual colonisation [234]. These metastatic stem cells will initiate colonisation following a latent period based upon the activation of signalling pathways, the tumour-initiating ability of metastatic cells and the presence of a supportive stromal microenvironment [163, 235, 236]. The role of HPSE in modulating the ECM would play a pivotal role within these distant metastatic sites which would facilitate the creation of a supportive microenvironment for metastatic colonisation. By virtue of its enzymatic and non-enzymatic functions, HPSE is therefore a key regulator of each step of the metastatic cascade.

#### 7. Genome instability and mutation: an enabling characteristic

Genomic instability is an inherent cause of most cancers, with compromised ‘caretaker’ and ‘guardian’ systems leading to malignant growth [2, 237, 238]. An aberrant ECM/TME is a critical enabler of this hallmark feature. The role of MMPs in cancer is not only limited to ECM remodelling, but also extends to causing tumour-initiating genetic alterations [239, 240]. For instance, the stromal expression of stromelysin-1 was shown to promote malignant changes in transgenic mouse mammary glands in conjunction with the upregulation of MMP-3 [241]. MMP-3 was shown to induce the expression of Ras-related C3 botulinum toxin substrate-1, causing the increase in reactive oxygen species (ROS), which in turn stimulated the Snail transcription factor expression, promoted EMT, caused oxidative DNA damage and led to genomic instability and malignant transformation of mouse mammary epithelial cells [242]. A similar ROS-induced tumorigenic function was suggested for MMP-9 in a mouse intestinal cancer model [243]. The overexpression of membrane type-1 MMP was shown to promote chromosomal instability, conferring tumorigenicity on normal cells [244, 245]. The expression of HPSE has been demonstrated to directly correlate with that of MMPs and to directly stimulate MMP-9 expression [182, 246, 247]. Thus, as a master regulator of MMPs, HPSE may play an indirect but critical role in achieving genomic instability through aberrant MMP expression.

HPSE also bypasses the tumour-suppressive roles of several genes, such as *PTEN*, *STAT3* and *TGF-β* [47, 62, 66]. It can be suggested therefore, that bypassing crucial protective roles of such genes amounts to an indirect promotion of genetic instability. Additionally, HPSE can localise to the nucleus, affecting gene expression [248]. This is thought to occur by passive transport, with gene expression achieved through nuclear-HS cleavage and the release of proteins such as FGF and topoisomerase-1 [249]. Translocation of HPSE to the nucleus has been shown to promote differentiation in human and mouse cancer cell lines [250, 251]. In a study of oesophageal squamous cell carcinoma patients, nuclear HPSE was shown to promote differentiation, but not proliferation [185]. However, in a study of head and neck squamous cell carcinoma patients, the nuclear localisation of HPSE was shown to indicate a favourable clinical outcome, in contrast to cytoplasmic localisation [252].

#### 8. Tumour-promoting inflammation: an enabling characteristic

Tumour-promoting inflammation is described as the ‘fuel that feeds the flames’ [253, 254]. Numerous immune cells have been shown to be intimately involved with the TME, promoting tumour progression [130, 134, 221, 255]. On account of the similarities between the tumour stroma and the inflammatory conditions in wounds, tumours have been described as ‘wounds that do not heal’ [256]. Additionally, infections have been suggested to be responsible for over 15% of malignancies, with inflammation playing a major role in infection-mediated cancer development [257, 258]. Although some infiltrating immune cells function in eliminating tumours, certain others promote tumour growth, resulting in a poor clinical outcome.

##### HS/HPSE-mediated immune cell migration and activation

Leukocyte migration into tissues is aided by HS and HPSE [259–261]. Leukocytes first establish adhesive interactions with endothelial cells leading to arrest, adhesion strengthening, crawling and the migration of cells through the vessel wall and into sites of inflammation. This is regulated by chemokines and the establishment of a chemokine gradient [262, 263]. HS has been shown to mediate cellular adhesion via cell surface molecules such as integrin and selectin, in both physiological and pathological conditions [264–267]. The adhesion of leukocytes to the endothelial wall is thus facilitated by HS, leading to cell arrest and the initiation of infiltration [268, 269]. A number of pro-inflammatory chemokines bind to HS, whose activity is thereby regulated [4, 270]. HS-mediated chemokine presentation plays a critical role in leukocyte recruitment, as demonstrated in an inducible mouse model deficient for exostoses-1, a key mediator of HS synthesis [271]. The enzymatic activity of HPSE liberates HS-bound chemokines, establishing a chemokine gradient and stimulating the recruitment of leukocytes [272]. HPSE-cleaved HS fragments were capable of stimulating the release of pro-inflammatory cytokines such as interleukin (IL)-1β, IL-6, IL-8, IL-10 and TNF through the toll-like receptor (TLR)-4 pathway in human peripheral blood mononuclear cells (PBMCs) and the release of IL-6, monocyte chemoattractant protein-1 and TNF in mouse splenocytes [273]. Fragmented HS has also been shown to activate dendritic cells (DCs) through TLR-4 stimulation, mediating an inflammatory response [274].

The activity of HPSE in promoting the migration of leukocytes was described even prior to the cloning of the enzyme [7, 135, 136]. This observation, coupled with that of HPSE-inhibiting substances such as heparin and HS-mimetics being capable of eliciting anti-inflammatory effects, establish the role of HPSE in a variety of inflammatory disorders [217, 275–277]. HPSE has been shown to affect several types of innate immune cells such as neutrophils, macrophages, DCs and mast cells that mediate both acute and chronic inflammatory responses [138, 278–282].

Although it was long-assumed that immune cells were the sole source of HPSE in inflammatory settings, numerous studies have demonstrated that epithelial cells also contribute to HPSE activity in conditions such as delayed-type hypersensitivity, ulcerative colitis, Crohn’s disease and acute lung injury following sepsis [279, 282–284]. In such conditions, HPSE was shown to be released upon the presence of inflammatory cytokines [279, 282, 284]. Furthermore, the nuclear localisation of HPSE was shown to induce endothelial cell gene expression and promote inflammation [285]. Nuclear HPSE was also shown to modify histone methylation patterns and promote an inflammatory T-cell phenotype [286]. More recently, the expression of HPSE by PBMCs, particularly T-cells, was shown to be stimulated by the secretion of HS-rich exosomes from tumour cells [287]. This in turn led to the release of exosomes rich in HPSE and HS, along with the release of HS chains by the activated T cells. The release of HS was proposed to induce HPSE expression in distant tumours and promote tumour growth. These data are consistent with earlier observations suggesting a crosstalk between cancer cells and PBMCs leading to HPSE overexpression by non-cancer cells which in turn promotes tumour growth [288].

##### HPSE in acute and chronic inflammation

Neutrophils are the major mediators of acute inflammation and related tissue injury [289]. In contrast to this traditional view, recent studies have shed light on the role of neutrophils in mediating chronic inflammation as well [290]. Cerulein-induced expression of HPSE expression has been shown to increase pancreatic cytokine (TNF-α, IL-6, etc.) and signalling molecule (phospho-STAT3) activity, along with enhanced oedema and inflammation marked by neutrophil infiltration, which ultimately led to acute pancreatitis [275]. In addition, the sepsis-induced upregulation of HPSE within the pulmonary microvasculature leads to the degradation of the endothelial glycocalyx, forming a HS-mediated chemotactic gradient, which recruits neutrophils and promotes lung tissue injury [279].

HPSE expression was observed in the colon of irritable bowel syndrome patients during both acute and chronic disease phases [282, 283]. Interestingly, the colonic epithelial cells were shown to be a major contributor of HPSE activity [283]. These interacted with macrophages in a HPSE-mediated manner to maintain a chronic inflammatory condition, which aided the formation of a tumour-promoting microenvironment with NF-κB signalling and induction of STAT3 expression [282]. HPSE was shown to generate a vicious cycle which promoted colitis and eventual colon cancer development by stimulating macrophages, which induced the production and activation of epithelial-HPSE via TNF-α and cathepsin-L. In a mouse model of allergic pulmonary cell recruitment, the lack of HPSE expression was shown to reduce eosinophil recruitment with no effect on neutrophils, resulting in a reduced allergen-induced bronchial hyper-responsiveness [277]. The same study demonstrated that lung specimens of patients with varying severity of chronic obstructive pulmonary disease showed an increase in HPSE expression. HPSE expression was also shown to promote macrophage activation, leading to TNF-α production in macrophages as well as in renal tissue and to enhance chronic inflammation associated with diabetic nephropathy [291]. In an interesting contrast, the overexpression of HPSE was shown to lead to aberrant neutrophil recruitment due to the HS-mediated chemokine gradient being disrupted on account of the enzymatic activity of HPSE [292].

##### HPSE in cancer-promoting inflammation

HPSE has been implicated in a number of inflammation-driven cancers. The progression of Barrett’s oesophagus to oesophageal carcinoma was associated with the gradual increase in HPSE activity [293]. Patients with hepatitis-C-related hepatocellular carcinoma showed a higher level of HPSE expression, which correlated with tumour angiogenesis and invasion [294]. In a clinical study, patient samples of chronic pancreatitis showed a high expression of HPSE, which increased further in cases of pancreatic cancer, resulting in poor post-operative survival [295]. Mice overexpressing HPSE showed accelerated progression of colitis to colonic tumours, with activated macrophages shown to induce HPSE expression in the colonic epithelial cells, promoting inflammation and cancer progression [282].

A large proportion of tumour-infiltrating immune cells are TAMs, which are key promoters of inflammation and contribute strongly to cancer progression [296, 297]. Activated macrophages express HPSE, aiding in ECM degradation [298]. In the aforementioned study of colon cancer, a cyclic relationship between HPSE and macrophage activation was reported [282]. Colonic epithelial cells expressing HPSE and mucosal macrophages interacted to maintain a chronic inflammatory condition, which aided the formation of a tumour-promoting microenvironment with NF-κB signalling and induction of STAT3 expression. HPSE was shown to generate a vicious cycle which promoted colitis and eventual colon cancer development by stimulating macrophages, which induced the production and activation of epithelial-HPSE via TNF-α and cathepsin-L. Recently, HPSE was shown to be pivotal in the activation and function of macrophages in the TME [10]. Using a genetic approach, mice lacking HPSE were shown to possess macrophages that expressed lower levels of cytokines such as TNF-α, IL-1β, IL-6 and IL-10. Macrophages lacking HPSE showed impaired phagocytic activity and reduced infiltrative capacity. Furthermore, these macrophages showed a significantly reduced expression of chemokine (C-X-C motif) ligand-2, which functions in attracting macrophages to sites of inflammation.

#### 9. Reprogramming energy metabolism: an emerging hallmark

Aberrant cancer-associated metabolism was a phenomenon first reported by Warburg, whereby cancer cells reprogram their glucose metabolism by limiting energy metabolism mainly to glycolysis, with increased glucose uptake and the production of lactate; a phenomenon referred to as the ‘Warburg effect’ [299–301]. The Warburg effect describes ‘aerobic glycolysis’, in which cancer cells preferentially employ a glycolytic energy metabolism pathway, even under aerobic conditions. Genetic studies suggest that the Warburg effect is indeed required for tumour growth, following decades of debate [302, 303].

HPSE affects glucose metabolism in several disease settings, which could suggest a similar role in cancer. The inhibition of HPSE in the apolipoprotein-E-deficient mouse model of atherosclerosis resulted in a marked reduction of serum glucose levels [304]. In type-2 diabetes mellitus patients, urine HPSE was shown to correlate with high blood glucose levels, indicating glucose-mediated HPSE expression and secretion, with follow up in vitro studies showing insulin-mediated HPSE secretion by human embryonic kidney cells in culture [305]. The interesting observation of HPSE improving glucose metabolism was made in a study of transgenic HPSE-overexpressing mice, with significant changes in pancreatic islet cell composition, structure, gene expression and the overall protective effect from streptozotocin-induced diabetes [306].

Tumours convert glucose or acetate into lipids, with tumour cells generating nearly all their cellular fatty acids via de novo synthesis [307, 308]. Fatty acids were shown to upregulate HPSE expression in endothelial cells through the Sp1 site within the *HPSE* gene promoter [309]. Further studies in endothelial cells showed that fatty acids caused the nuclear translocation of HPSE, leading to the regulation of genes related to glycolysis and the accumulation of lactate, a vital fuel source and regulator in cancer progression [285]. Additionally, the PI3K signalling pathway has been shown to promote glycolysis and the Warburg effect in cancers [310, 311]. HPSE has been shown to promote PI3K signalling, which suggests a role in promoting the Warburg effect [62].

Tumours have adapted survival mechanisms to overcome hypoxia which includes the upregulation of HIF transcription factors [139–141, 145, 312]. HIF-1-mediated gene expression has been shown to promote the Warburg effect in cancers by directing the cellular energy pathway towards glycolysis [313, 314]. The upregulation of HPSE in hypoxic conditions and the HPSE-mediated upregulation of HIFs may therefore suggest a role in hypoxia-mediated modifications to cancer metabolism [146, 147, 149, 315, 316].

#### 10. Evading immune destruction: an emerging hallmark

It is now known that cancer-associated immune cells can be either detrimental or beneficial to its progression [317, 318]. Macrophages form a significant portion of tumour-associated immune cells, with HPSE playing a key role in their activation and function [10, 296]. Macrophages are capable of promoting tumours through the induction of immunosuppression [297]. For example, macrophages express human leukocyte antigen (HLA) molecules such as HLA-C, HLA-E and HLA-G that are capable of inhibiting NK cells and certain activated T cell subsets [319]. The programmed cell death protein-1 (PD-1) and programmed death ligand-1 (PDL-1) pathway is a potent target in cancer therapy [320–322]. PD-1 expression by TAMs has been shown to reduce anti-tumour immunity and to promote the pro-tumorigenic M2 macrophage phenotype [323].

HPSE was shown to regulate the secretion of cytokines such as TNF-α, IL-1β, IL-10 and IL-6 by macrophages which have demonstrated functions in promoting an immunosuppressive TME [10]. Macrophages also secrete chemokines that suppress CD4^+^ and CD8^+^ T cell function by the recruitment of regulatory T (T_reg_) cells, with the infiltration of the TME by T_reg_ cells generally associated with a poor clinical prognosis [324, 325]. The regulation of TAMs by HPSE suggests its indirect role in the recruitment of T_reg_ cells to the TME.

### HPSE in the TME

**Fig. 2:**
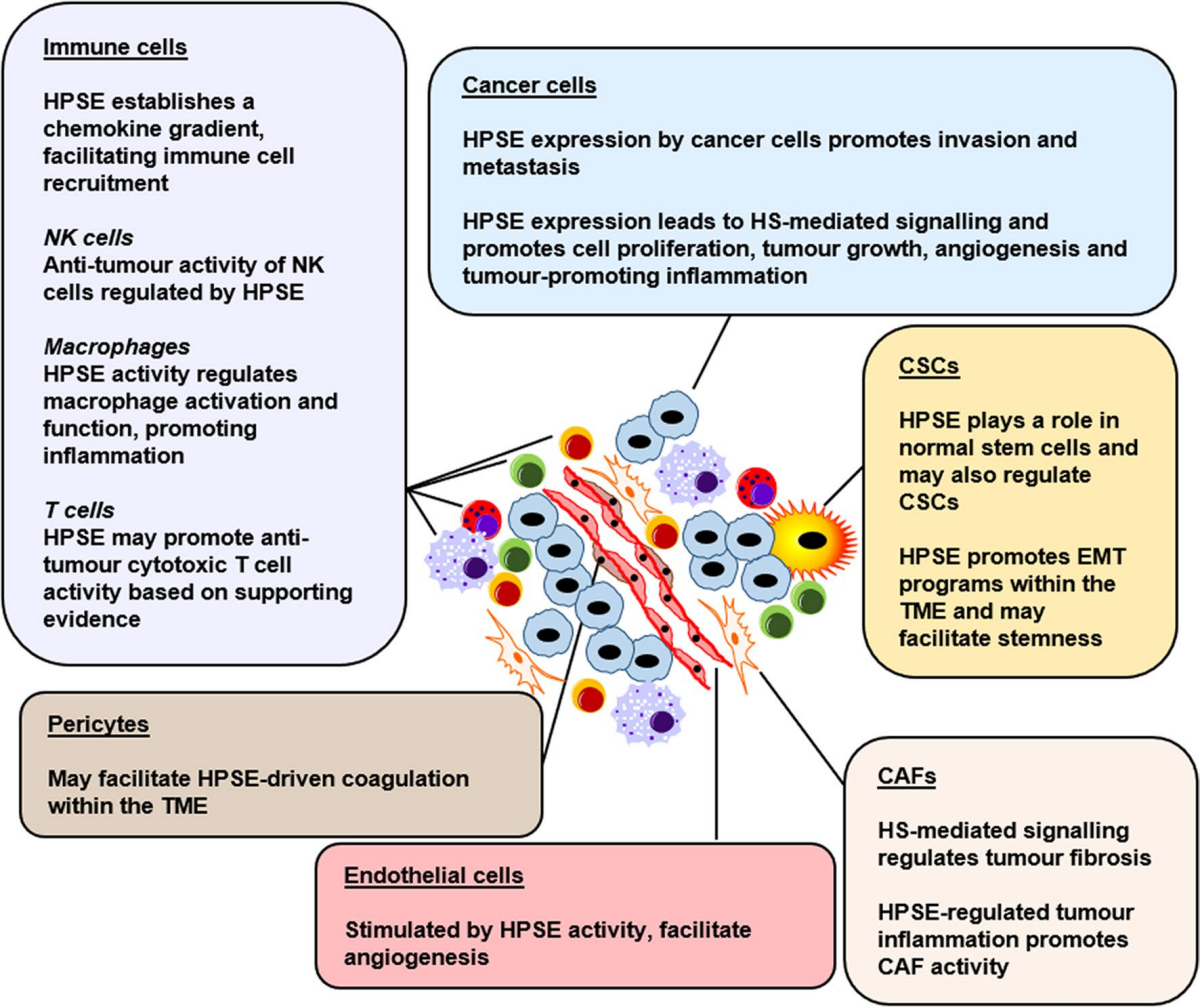
HPSE regulates multiple components within the TME. The TME is composed of numerous cell types. HPSE is a key regulator of the major components of the TME, which promotes their pro-tumorigenic properties. Critical anti-tumour properties within the TME are also regulated by HPSE

Tumours are heterogeneous entities and are comprised of a number of different cell types, both cancerous and otherwise, collectively forming the TME [231]. Solid tumours present dynamic ecosystems, with a level of organisational complexity at times rivalling that of normal tissues [326]. Constant crosstalk between cancer cells and their stromal counterparts maintains a vital tumour-promoting line of communication. HPSE regulates key components of the TME (Fig. 2), discussed as follows.

#### Cancer cells and CSCs

Cancer cells are the fundamental building units of a tumour and carry defining genetic properties [2]. HPSE expressed by cancer cells promotes a number of key hallmark features as described previously, such as proliferation, inflammation, invasion and metastasis and angiogenesis. All human cancers are known to overexpress HPSE. Multiple clinical studies and patient sample analyses have demonstrated this aberrant expression as well as the correlating poor clinical prognosis in a variety of malignancies including breast, prostate, lung, pancreatic, head and neck, oral, colorectal, gastric, thyroid, liver, bladder, and cervical cancer as well as melanoma, lymphoma and leukaemia [118, 183, 186, 252, 294, 295, 327–344].

More recent observations have indicated the presence of a second subset of cancer cells within the TME, the CSCs, with the ability to give rise to new tumours [2]. With the discovery of genetic mutations as the major cause of cancers, the clonal evolution concept was proposed by Nowell, stating that most neoplasms had a single cell of origin with tumour progression resulting from acquired genetic variability within the original clone, subsequently allowing the selection of aggressive cancer cell sublines [345]. The current CSC model is based on the premises that tumour heterogeneity arises from its hierarchical organisation driven by rare CSCs whose identity is hardwired, and that CSCs are largely responsible for tumour relapse by virtue of their resistance to standard therapies [106].

Several in vivo studies have demonstrated the role of HPSE in normal stem cell function. HPSE was shown to affect basic hematopoietic stem and progenitor cells as well as the bone marrow environment [346]. Loss-of function studies employing HPSE inhibitors demonstrated that the enzymatic activity of HPSE was key in proliferation and colony formation efficiency of mouse bone marrow-derived mesenchymal stem cells [347]. A growth advantage was imparted upon HPSE-overexpressing mouse embryonic stem cells, which formed larger teratomas when inoculated in vivo [348]. Furthermore, in a study to determine the therapeutic potential of hypoxic preconditioning mesenchymal stem cells (HPC-MSCs), mice injected with HPSE overexpressing HPC-MSCs showed enhanced blood flow recovery on account of the pro-angiogenic properties of HPSE [316]. These observations suggest that HPSE may play a role in CSCs and warrants further investigation.

EMT features have been shown to play a direct role in imparting cellular stemness, with a study demonstrating the expression of EMT markers in normal mammary gland stem cells as well as mammary CSCs of both human and mouse origin [349]. Furthermore, the induction of EMT in human breast cancer cells was shown to impart stem-like properties upon them [350]. These and other studies have shed light on the unexpected observation that EMT programs impart stemness in both normal and neoplastic cells [351, 352]. As discussed, HPSE promotes EMT features in cancer cells [27, 47, 170–175, 353]. Therefore, EMT not only aids metastatic dissemination, but may also play a key role in the generation of a reservoir of CSCs able to continuously seed tumours and ultimately lead to therapy resistance and relapse. Although there is a lack of studies directly implicating HPSE in the generation of CSCs, this may be achieved indirectly through the promotion of EMT programs within the TME.

#### Endothelial cells and pericytes

Endothelial cells found in the TME are fundamentally different to those found in normal, healthy tissues. For instance, these cells tend to be cytogenetically abnormal [354, 355]. The gene expression profile, angiogenic properties and the growth factor responses of these endothelial cells also drastically differ from those in normal tissue [356–359]. Furthermore, tumour-associated endothelial cells exhibit aberrant chemotherapeutic responses, complicating disease treatment [360–362].

Human vascular endothelial cells were shown to produce active HPSE, released at times of cellular injury and death [119]. Inflammatory cytokines such as TNF-α and IL-1β were demonstrated to promote HPSE expression in endothelial cells [309]. The TME can harbour an inflammatory environment which may stimulate HPSE production by endothelial cells, causing the remodelling of the sub-endothelial matrix, thus leading to enhanced cell proliferation and angiogenesis. The crosstalk between cancer cells and endothelial cells is regulated by HPSE, leading to tumour angiogenesis.

Pericytes, along with endothelial cells, are structural components of blood vessels found embedded within the microvessel BM and play a key role in TME maintenance and regulation [363]. Multiple studies have described the aberrant organisation of pericytes within tumour-associated blood vessels, the pericyte-mediated effects on BM organisation and endothelial cell function as well as their overall effects on clinical outcomes [364–369]. Targeting pericytes has been suggested as a novel therapeutic option in the treatment of cancers [370].

The precise role of HPSE in connection with pericytes within the TME is yet to be conclusively elucidated. However, pericytes may be key in HPSE-driven coagulation in the TME. TF, crucial in the coagulation cascade, is primarily expressed by pericytes and generally not by endothelial cells [371]. HPSE has been shown to participate in the coagulation cascade as a co-factor of TF activity [372]. A number of cancers have been identified to possess a pro-thrombotic state, which raises the possibility of a pericyte-initiated mechanism of tumour-promoting coagulation, aided by HPSE [373, 374]. Interestingly, Hunter et al. reported that the deletion of HPSE in mice led to increased angiogenesis and pericyte coverage in pancreatic neuroendocrine tumours, suggesting a HPSE-dependent organisation of pericytes [157].

#### Cancer-associated fibroblasts (CAFs)

Fibroblasts are capable of producing ECM components such as proteoglycans, laminin, glycosaminoglycans, collagen, glycoproteins, hyaluronic acid and HS, aiding in wound healing [375–377]. Fibroblasts are also capable of modifying the ECM through the expression of MMPs in both physiological and malignant conditions [378–380]. It is indeed this wound healing capability of fibroblasts that leads to pathologic fibrosis found in a number of organs and tissues such as eye, skin, heart, lungs, liver, kidney and pancreas [381]. Fibrosis is also a feature of solid tumours, associated with major ECM modifications in the TME, which ultimately promotes metastasis [382]. However, the precise role of fibrosis in cancer is currently debated, with data emerging to suggest a paradoxical nature of fibrosis playing both positive and negative regulatory roles [383].

Pathological fibrosis is dependent on growth factor signalling [375]. Clinical data has demonstrated that the inhibition of FGF, PDGF and VEGF as well as multiple tyrosine kinases that are critical in promoting fibrosis lead to a favourable patient outcome [384]. TGF-β is considered the master regulator of fibrosis and is potent in activated fibroblast recruitment in cancers and several other disease settings [385–389]. Studies on several pathological conditions have shed light on the role of HPSE in fibrosis [390]. HPSE has been shown to play a key role in the EMT transition of proximal tubular epithelial cells to myofibroblasts in renal fibrosis by regulating HS-mediated FGF signalling [353]. Additionally, HPSE has been suggested as a master regulator of TGF-β signalling, leading to the conversion of tubular cells to myofibroblasts by enhancing EMT [47]. In a mouse model of diabetes nephropathy, mice lacking HPSE experienced significantly reduced interstitial fibrosis [391]. Furthermore, dysregulated paracrine and autocrine signalling has been shown to convert hepatic stellate cells into myofibroblasts, leading to liver fibrosis in a process largely mediated by macrophage-derived HPSE [281]. Lastly, in a mouse model of pulmonary fibrosis, HPSE released by activated fibroblasts enhanced TGF-β signalling, leading to the progression of bronchiolitis obliterans syndrome [392].

In the TME, crosstalk between cancer cells and the CAFs is likely mediated by HPSE expressed mainly by cancer cells and tumour-infiltrating immune cells. The enzymatic activity of HPSE liberates a number of HSBPs including TGF-β, FGF, PDGF and VEGF, which may directly contribute to fibroblast recruitment and activation in the TME, resulting in cancer fibrosis. The inflammatory nature of the TME can also be modified by fibroblast activity, where NF-κB signalling activation in fibroblasts leads to a tumour-promoting inflammatory signature, resulting in increased recruitment of macrophages and angiogenesis [393]. This education of fibroblasts is thought to be initially mediated by tumour-associated immune cells, mainly TAMs. HPSE is a potent regulator of tumour inflammation, especially mediating TAM activity [10]. This suggests an indirect role of HPSE in the modification of fibroblast activity in the TME through promoting immune cell recruitment and activation. Primary human fibroblasts have been shown to be capable of converting enzymatically inactive pre-HPSE into its active form [394]. This modulatory capability may contribute to upregulated HPSE activity within the TME, enhancing tumour growth. HPSE is also highly expressed in the accompanying stromal fibroblasts in colon carcinoma metastases [331]. This suggests a role in CAF-derived HPSE in promoting colonisation by modifying the metastatic niche.

#### Immune cells

**Fig. 3:**
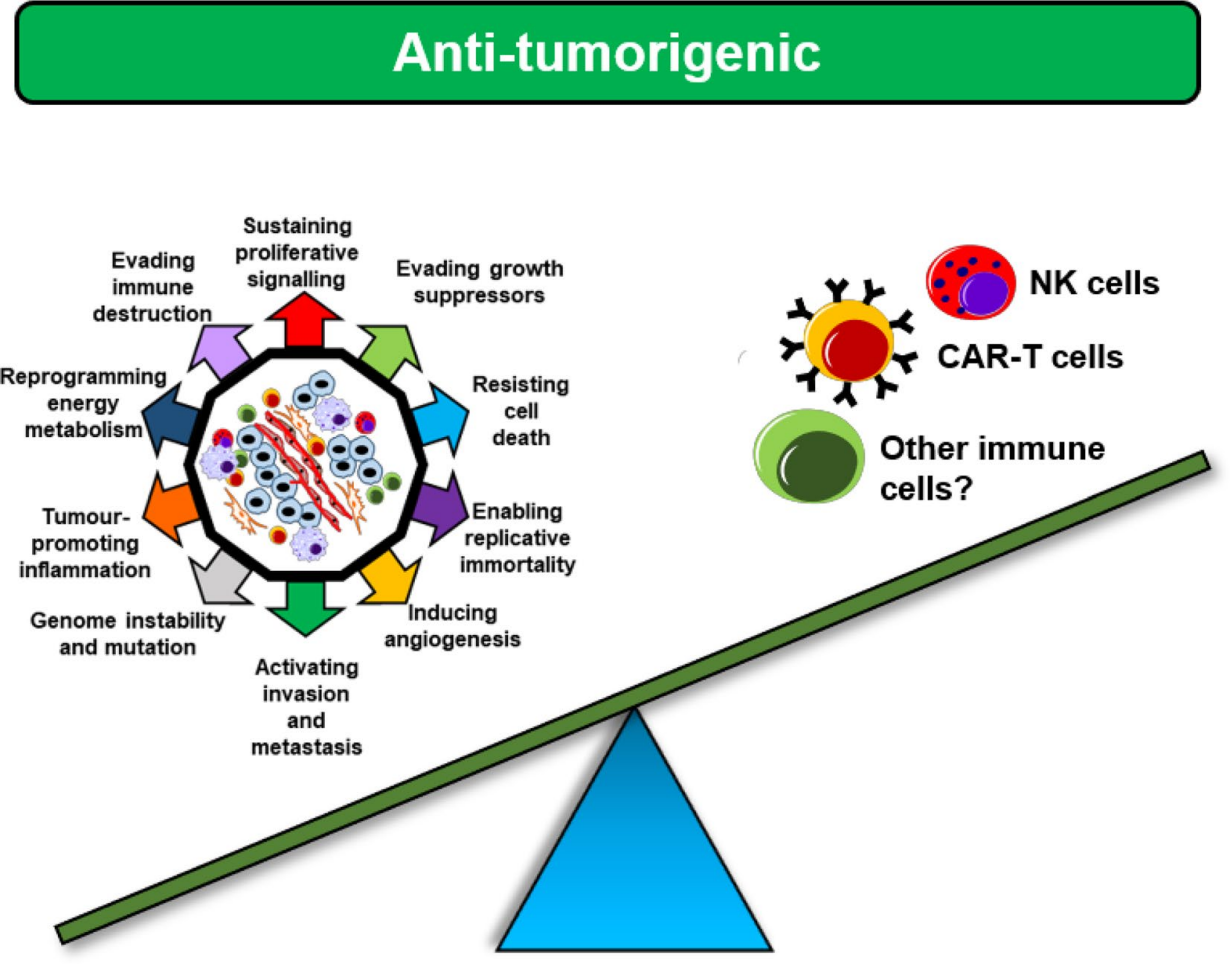
HPSE demonstrates anti-tumorigenic properties. Long assumed to be a promoter of tumorigenicity, HPSE has recently been shown to enable tumour immunity through regulating NK cell and CAR-T cell activity. Similar properties may be bestowed by HPSE upon other anti-tumour immune cell types as well

Previous sections of this review explored the mechanisms by which HPSE regulates immune cell recruitment to the TME, leading to tumour progression. Based on these observations, HPSE could be assigned a predominantly tumour-promoting role. However, recently published data challenge this notion (Fig. 3).

In parallel to the well characterised phenomenon of immune cells promoting tumour growth, it is also understood that the immune system plays a critical role in preventing the establishment and the progression of cancers [395]. Amongst the large array of tumour-associated immune cells, NK cells have emerged as a potent safeguard against tumour and metastatic growth and is a key player in tumour immunosurveillance [396]. This has led to the recent interest in the promise of NK cells in directed tumour immunotherapy [397].

In contrast to previous observations of tumour-associated immune cells promoting cancer progression in a HPSE-dependent manner, Putz et al. recently reported that HPSE was vital in NK cell-mediated anti-tumour activity [10, 11]. A study involving human and mouse NK cells demonstrated that HPSE expression was significantly upregulated upon NK cell activation and that mice lacking NK cell-specific HPSE expression exhibited impaired invasion and tumour surveillance. The in vivo growth of tumours was also significantly enhanced with the lack of NK cell-HPSE activity. Additionally, the efficacy of immunotherapy was drastically reduced in tumour-bearing mice lacking NK cell-specific HPSE. This pivotal study has shed light on a previously unknown role of HPSE in regulating NK cell-mediated tumour immunosurveillance.

Cytotoxic lymphocytes, along with NK cells have also emerged as key regulators of anti-tumour immunity [398]. This protective function has resulted in the engineering of CAR-T cells in an effort to provide targeted, highly effective cancer therapy [399]. In a recent landmark study, an increase in HPSE activity in CAR-T cells was shown to significantly enhance tumour invasion and anti-tumour immunity [400]. Even though no direct evidence linking HPSE expression and T cells in a physiological anti-tumour setting has yet been reported, such a relationship can be strongly suggested based on these observations.

#### A dual role of HPSE within the TME?

Cancers are driven by complex signalling networks within the TME, initiated by neoplastic cells that promotes the recruitment and activation of the cancer-associated stroma to support tumour growth and metastasis [2, 401]. This signalling further extends to the modulation of the metastatic niche by the primary malignancy. As this review has highlighted, HPSE-mediated crosstalk amongst the various components of the TME promotes tumour maintenance and progression. HPSE continues to generate significant interest as a potential therapeutic target due to its multiple roles in tumour progression [402]. As such, several inhibitors have progressed to human clinical trials, with many others in various stages of development [32, 125–127, 193, 403–406].

**Fig. 4:**
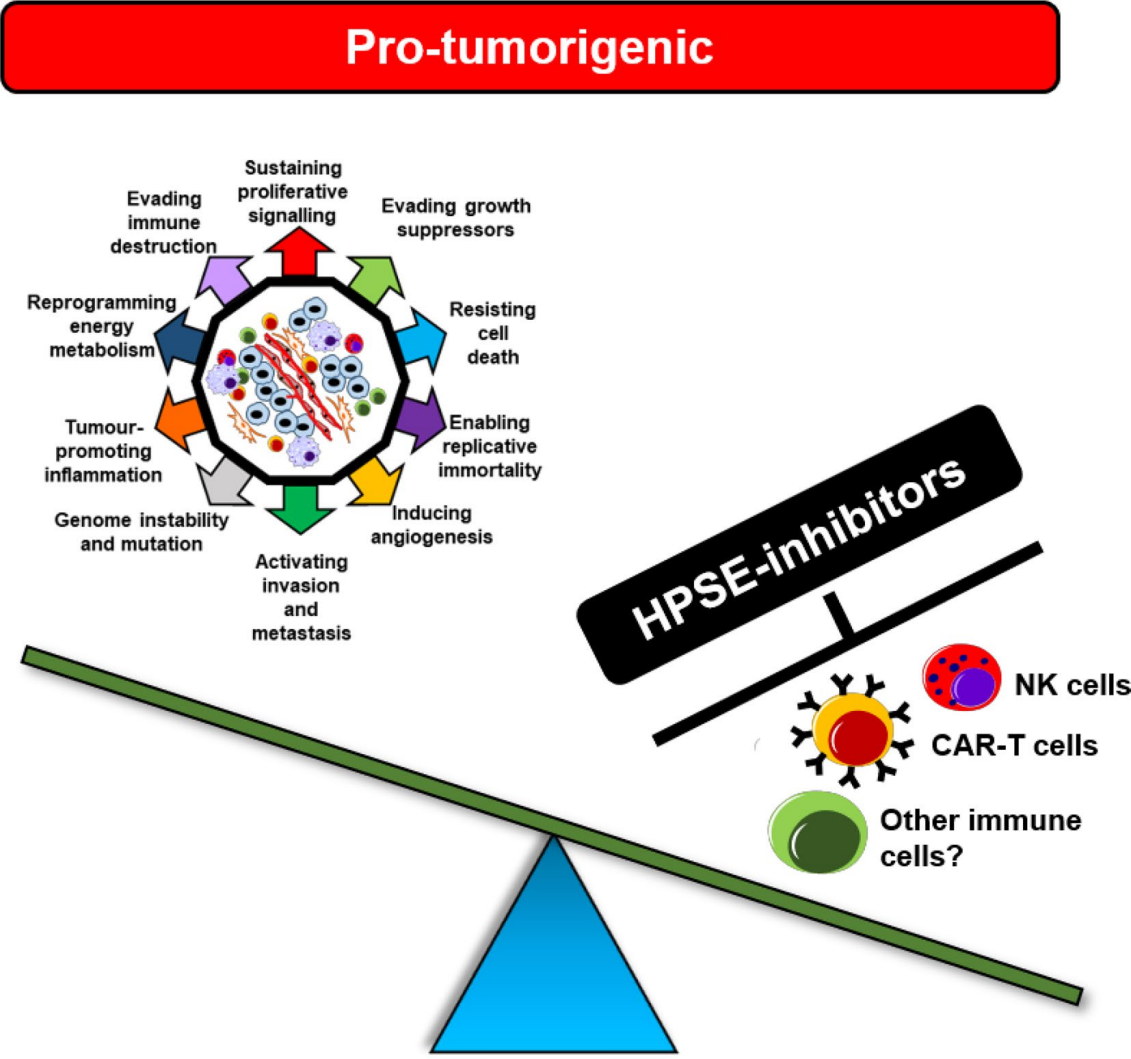
Targeting HPSE within the TME may promote tumour growth. The indiscriminate targeting of HPSE within the TME may compromise tumour immunity by inhibiting immune cells responsible for tumour immunosurveillance and anti-tumour activity. It is therefore important that the precise role of HPSE within each tumour setting is thoroughly analysed prior to the administration of HPSE inhibitors

However, recent data regarding HPSE-mediated tumour immunity raises the possibility of a dual role of HPSE within the TME in both promoting and inhibiting tumour growth. In light of these contradictory findings, a critical question ought to be raised whether targeting HPSE in the TME may prove detrimental or beneficial to a patient. As the complexity of the role of HPSE in cancer continues to unravel, it is now clear that a one-size-fits-all approach may not be ideal in certain tumour settings. Indeed, HPSE inhibitors may result in more harm than benefit in some cancers (Fig. 4) and may explain why several human trials in the past experienced failures and have since been discontinued.

The development and first human cancer trials of MMP inhibitors provide valuable insights into the complexity of targeting TME components with proven contradictory roles. Early broad-spectrum MMP inhibitors suffered multiple failures, with their administration resulting in the worsening of tumour progression by the unintended but unavoidable blocking of MMPs with anti-tumour activity and those crucial in maintaining normal physiology [407, 408]. This is testament to the risk of indiscriminately targeting ECM-modifying enzymes in the TME. Therefore, it is vital that the precise role of HPSE in a given tumour setting is elucidated, with its pro and anti-tumour roles thoroughly addressed prior to the use of HPSE inhibitors.

## Conclusion

The definition of the hallmarks of cancer has revolutionised cancer research, with the ECM having revealed itself not as a mere bystander, but as a major regulator of malignant disease. ECM-modifying enzymes such as HPSE have therefore gained significant interest as therapeutic targets.

With its roles in both the maintenance of normal physiology and the promotion of several pathologies, HPSE has emerged as a ‘jack-of-all-trades’. It was this notion that spurred this review as it was clear that through its multi-faceted nature, HPSE may be a potent driver of all hallmarks of cancer. Despite several decades of research, our understanding of HPSE and its many functions continues to evolve. Adding to this complexity are the recent findings that HPSE plays a role in preventing tumours through activating cells of the innate immune system. With the current trend towards the discovery and clinical trials of novel HPSE inhibitors, this contradictory role of HPSE in cancer must be addressed. Therefore, despite our current knowledge, much work is needed to navigate the grey areas created by recent studies. HPSE may very well be revealed to not be a ‘holy grail’ target within the TME, but a highly complex, unpredictable and underestimated entity.

## Data Availability

Not applicable.

## Abbreviations

Akt: Protein kinase-B
BM: Basement membrane
CAF: Cancer-associated fibroblast
CAR-T: Chimeric antigen receptor-T cell
CCL2: Chemokine (C–C motif) ligand-2
CSC: Cancer stem cell
CTC: Circulating tumour cell
DC: Dendritic cell
DNA: Deoxyribonucleic acid
ECM: Extracellular matrix
EGF: Epidermal growth factor
EGFR: Epidermal growth factor receptor
EMT: Epithelial-mesenchymal transition
FGF: Fibroblast growth factor
FGFR: Fibroblast growth factor receptor
HGF: Hepatocyte growth factor
HIF: Hypoxia-inducible factor
HLA: Human leukocyte antigen
HPC-MSC: Hypoxic preconditioning mesenchymal stem cell
HPSE: Heparanase
HS: Heparan sulphate
HSBP: Heparan sulphate-binding protein
HSPG: Heparan sulphate proteoglycan
hTERT: Human telomerase reverse transcriptase
IL: Interleukin
MMP: Matrix metalloproteinase
mTOR1: Mammalian target of rapamycin-1
NF-κB: Nuclear factor kappa-light-chain-enhancer of activated B cells
NK: Natural killer
PBMC: Peripheral blood mononuclear cell
PD-1: Programmed cell death protein-1
PDGF: Platelet-derived growth factor
PDL-1: Programmed death ligand-1
PI3K: Phosphatidylinositol-3-kinase
PTEN: Phosphatase and tensin homolog
ROS: Reactive oxygen species
STAT: Signal transducer and activator of transcription
TAM: Tumour-associated macrophage
TEM: Trans-endothelial migration
TF: Tissue factor
TGF-β: Transforming growth factor-beta
TLR-4: Toll-like receptor-4
TME: Tumour microenvironment
TNF: Tumour necrosis factor
T_reg_: Regulatory-T
VEGF: Vascular endothelial growth factor
